# Effects of a Dog-Assisted Animal-Assisted Therapy on Behavioural Functioning in Preschool and Primary School Children: A Randomised Controlled Study

**DOI:** 10.3390/children13070924

**Published:** 2026-07-14

**Authors:** Beáta Erika Nagy, Éva Zita Balogh, Karolina Eszter Kovács

**Affiliations:** 1 Pediatric Psychology and Psychosomatic Unit, Institute of Pediatrics, Pediatric Rehabilitation, Faculty of Medicine, University of Debrecen, 4032 Debrecen, Hungary; drbeatanagy@gmail.com; 2 Doctoral Program on Psychology, Doctoral School of Humanities, University of Debrecen, 4032 Debrecen, Hungary; evezita@yahoo.com; 3 Institute of Psychology, Faculty of Arts, University of Debrecen, 4032 Debrecen, Hungary

**Keywords:** animal-assisted therapy, dog-assisted therapy, children with special needs, behavioural problems, hyperactivity

## Abstract

Background: Animal-assisted therapy (AAT) is a targeted intervention that supports participants’ development by involving animals in the therapeutic process. This study aimed to investigate the effectiveness of AAT with preschool and primary school children, including children with special educational needs (SEN). Methods: A total of 201 children (114 experimental, 87 control; including children with and without special educational needs) participated in the study. Behavioural functioning was assessed using the parent-rated Conners questionnaire and the Strengths and Difficulties Questionnaire (SDQ). Data were analysed using the Wilcoxon test and repeated measures ANOVA. Results: Mixed-design repeated measures ANOVA showed no significant Group × Time interaction for the primary behavioural outcomes, indicating that behavioural changes over time did not differ significantly between the intervention and control groups. Within-group analyses showed improvements in parent-reported oppositional behaviour, cognitive problems/inattention, hyperactivity, and conduct problems in the intervention group; however, similar improvements were also observed for some outcomes in the control group. Conclusions: Although behavioural improvements were observed over time, the intervention did not result in significantly greater improvements than those observed in the control group. Dog-assisted AAT may provide a supportive therapeutic environment that facilitates engagement and behavioural practice; however, further adequately powered randomised controlled trials are needed to determine its effectiveness.

## 1. Introduction

Animal-assisted therapy (AAT) is a health intervention meant to improve physical, social, emotional or cognitive functioning, with animals as an integral part of the treatment [1]. Historically, William Tuke was the first to document the use of AAT in the eighteenth century. He believed that people in mental hospitals were subjected to inhumane treatment and helped to improve their lives by encouraging them to care for animals [2]. Boris Levinson [3] recorded the therapeutic benefits for individuals who came into contact with pets and applied AAT to psychological practice. AAT is based to some extent on the theory of biophilia, a hypothesis first introduced by Wilson [4]. The hypothesis explains that all humans are innately connected to nature and other living things.

AAT uses the human–animal bond as an integral part of the treatment process in targeted interventions. Working animals and their handlers must be trained and meet a number of specific criteria. A licenced therapist, working within the framework of professional practice, sets therapeutic goals, guides patient–animal interaction, measures the process required to achieve therapeutic goals, and evaluates the process [5,6]. Therefore, AAT can be seen as a complement to an existing therapy. The therapist can incorporate the animal into any professional therapy style. AAT can be either direct or nondirective in terms of the relationship. AAT consultations can be incorporated into individual or group therapy. They can be used in an extensive range of settings, with various age groups and individuals with varying abilities. The persons conducting AAT should have appropriate training [7].

Several studies have shown that dog-assisted therapy has a significant positive impact on the lives of children with developmental disabilities, particularly in terms of their ability to concentrate and their motivation to work. The presence of dogs during the therapy process provides emotional support and creates a sense of security for the children [8,9]. This atmosphere allows them to be more open, express themselves more efficiently, and participate actively in learning. Interaction with dogs helps them develop their social skills and plays a key role in maintaining attention and concentration [1].

### 1.1. Physiological Effects of AAT

Interacting with dogs can be associated with physiological changes such as reduced stress levels and improved emotional well-being. The presence of a dog and the bond that develops between them helps to lower blood pressure, reduce stress levels and induce a general sense of relaxation. This process is supported by research that shows that people’s moods can be significantly improved and the experience of social interaction enriched when interacting with animals [10].

Research by Jorge and colleagues [11] has shown that dog-assisted therapy significantly impacts children’s motor development. During therapy, children feel safe and happy in the company of dogs and develop motor skills by practising different movement patterns. In particular, there are positive changes in balance, motor planning and spatial orientation, which are key to children’s physical development.

AAT plays an important role in supporting people with disabilities, especially those with intellectual disabilities. In this therapy, dogs are companions and active participants in the rehabilitation process. Dog-assisted therapy programmes encourage physical activity and develop emotional intelligence and cognitive functions. People with intellectual disabilities often experience the joy and satisfaction of playing or walking with a dog. These positive experiences not only enrich their psyche but also contribute to their development, as the trust and bonding that develop during therapy enhance self-esteem and the development of social relationships [12,13].

Our systematic analyses of the physiological and psychological effects of AAT [14,15] and the literature provide evidence of the positive effects of animal-assisted programmes on physiological health. Positive and supportive effects on both the nervous and motor systems have been demonstrated compared to control groups receiving standard therapy. The magnitude of the effect may vary depending on the type, nature, severity and comorbidity of the disorder. Abadi et al. [16] have shown that AAT improves the development of children with autism, and the results of Calcaterra et al. [17] have confirmed that AAT can have a significant positive effect on recovery from surgery. The new models support the complex management of disorders, including their physiological and psychological aspects [18].

### 1.2. Psychological Effects of AAT

Beyond its physiological and emotional benefits, AAT has increasingly been investigated as a supportive intervention for behavioural and cognitive difficulties in children. Particular attention has been given to children presenting symptoms of attention-deficit/hyperactivity disorder (ADHD), executive functioning deficits, and difficulties in behavioural self-regulation. These challenges often include problems with sustaining attention, inhibiting impulsive responses, following instructions, and adapting behaviour to situational demands. Several studies suggest that interaction with therapy animals may support behavioural regulation through multiple mechanisms. Therapy animals can increase children’s motivation to participate in structured activities, provide immediate and non-judgmental feedback, and facilitate engagement in tasks requiring sustained attention and self-control. Busch et al. [19] argued that AAT may be particularly beneficial for children with ADHD because the presence of animals can reduce stress, improve attention regulation, and promote adaptive behavioural responses. Similarly, Carlisle et al. [20] highlighted the potential role of AAT in supporting children with executive function deficits, including difficulties related to inhibitory control, working memory, and self-monitoring.

AAT may also contribute to the reduction of disruptive and oppositional behaviours. Participation in structured activities involving therapy animals often requires children to follow rules, wait for their turn, regulate impulses, and cooperate with both peers and therapists. These experiences may strengthen behavioural self-regulation and social competence. Furthermore, animal-assisted counselling programmes have been associated with improvements in emotional regulation and behavioural adjustment among children and adolescents [21]. A systematic review of classroom-based AAT also reported positive effects on attention, engagement, and classroom behaviour [22,23].

Despite these promising findings, relatively few controlled studies have examined the effects of dog-assisted therapy on behavioural functioning among preschool and primary school children, particularly those with special educational needs (SEN). Given that difficulties in attention regulation, hyperactivity, oppositional behaviour, and conduct problems are among the most frequently reported challenges in this population, these domains represent important targets for intervention. Therefore, the present study focused specifically on behavioural and cognitive outcomes assessed through parent-reported measures of attention difficulties, hyperactivity, oppositional behaviour, and behavioural problems.

### 1.3. Aims and Research Questions

Previous studies suggest that interactions with therapy dogs may enhance attention [24,25], increase motivation to engage in structured tasks [26,27], promote compliance with instructions [28,29], and support emotional and behavioural self-regulation [24,30]. These mechanisms are particularly relevant for children who exhibit hyperactivity, impulsivity, attention difficulties, and oppositional behaviours, which are among the most common challenges observed in children with special educational needs. Consequently, behavioural functioning represents a particularly relevant outcome domain when evaluating the effectiveness of dog-assisted interventions. Therefore, the present study focused on behavioural and cognitive outcomes assessed through the Conners Child Behaviour Questionnaire and the Strengths and Difficulties Questionnaire (SDQ), rather than on motor or physiological outcomes.

Children with special educational needs often experience greater difficulties in attention regulation, behavioural self-regulation, impulse control, and social adaptation than their typically developing peers [31]. Because AAT provides structured opportunities for practising attention, behavioural control, cooperation, and task engagement, children with SEN may have greater potential for improvement in these domains. Previous studies have suggested that children with developmental, behavioural, and learning difficulties often derive particular benefit from AAT due to the motivational, supportive, and non-judgemental nature of interactions with therapy animals.

The aim of this research was to investigate the effectiveness of AAT in preschool and primary school children, with a particular focus on reducing behavioural and cognitive problems, hyperactivity and attention deficit disorder. The research focused on the therapeutic development of children with special educational needs (SEN). Our research question was: To what extent does AAT contribute to the reduction of behavioural, cognitive and emotional problems and the development of social skills in preschool children? Our main research hypothesis was that AAT would result in significant improvements in children’s behavioural and cognitive functioning, particularly in the reduction of oppositional behaviour, hyperactivity and attention deficit symptoms. In addition, we hypothesised that AAT would result in more effective improvements in children with SEN than in typically developing children.

## 2. Methods

A total of 201 children were recruited for the study. After screening for eligibility criteria, participants were randomly assigned to the experimental group (n = 114) or the control group (n = 87). All participants completed the baseline assessment. Post-intervention data were collected after the completion of the ten-session intervention period. The analyses were conducted on all participants with available pre- and post-intervention data.

### 2.1. Participants

The target group of the research was preschool and primary school children. The research was conducted with children with special educational needs and typically developing children. Inclusion criteria for the SEN group were the presence of an officially documented special educational need and an age between 6 and 10 years. Preschool and early primary school children were selected because this developmental period is particularly important for the emergence and consolidation of behavioural self-regulation, attention control, and social functioning. Difficulties related to hyperactivity, impulsivity, oppositional behaviour, and attention regulation often become more apparent during the transition to formal educational settings, where children are expected to follow rules, sustain attention, cooperate with peers, and engage in structured learning activities. Consequently, this age range represents a particularly relevant period for evaluating interventions aimed at improving behavioural and cognitive functioning. Exclusion criteria included severe autism spectrum disorder that prevented participation in group-based activities, moderate to severe intellectual disability, severe sensory impairments, and behavioural difficulties that made participation unsafe. Children receiving other educational or developmental services as part of their regular care were not excluded; however, no additional animal-assisted interventions were provided during the study period. Although our primary interest was the effectiveness of AAT, we considered a control group necessary to account for developmental changes, maturation effects, repeated testing effects, and other external influences that may occur during the intervention period.

**Figure 1:**
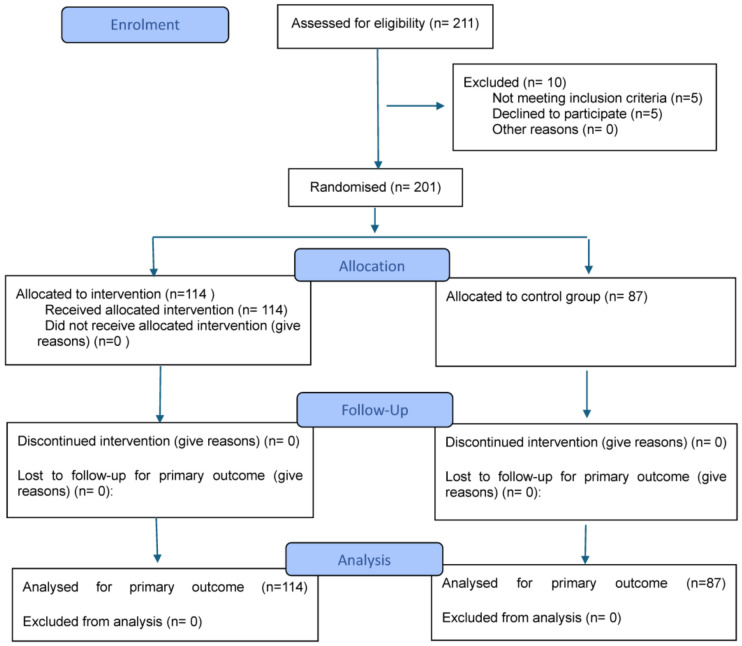
CONSORT 2025 Flow diagram of the progress through the phases of a randomised trial of two groups (that is, enrolment, intervention allocation, follow-up, and data analysis).

A total of 201 children were recruited for the study. The sample was drawn in Hungary and Romania. A study group (N = 114) and a control group (N = 87) were created. The study group included 77 typically developing children and 37 children with SEN, while the control group included 32 children with SEN and 55 typically developing children. The control group design provided an opportunity to measure the experimental conditions and effects more accurately. The control group consisted of children who did not participate in the intervention, so a comparison allowed for a valid evaluation of the impact of the programme. The groups were designed using a randomised controlled trial (RCT), i.e., children were randomly assigned to each group. The control group allowed the comparison of outcomes alongside the AAT (Figure 1). Simple randomisation was applied without blocking; therefore, minor differences in group sizes occurred by chance.

Children were classified as having special educational needs (SEN) based on an official diagnosis documented in educational or medical records. Because the study was conducted in Hungary and Romania, the classification followed the national regulations of the respective countries. In both countries, SEN status is established through a multidisciplinary assessment involving educational psychologists, special educators, physicians, and other relevant specialists. The diagnostic process typically includes cognitive, behavioural, developmental, and educational evaluations, followed by an official expert opinion determining eligibility for special educational support services.

The SEN group included children with documented developmental, learning, attention, behavioural, or communication difficulties requiring special educational support. The most common categories included attention-deficit/hyperactivity disorder (ADHD), specific learning difficulties, developmental disorders, and mild neurodevelopmental impairments. Children with severe autism spectrum disorder, moderate-to-severe intellectual disability, or conditions preventing participation in group activities were excluded from the study. Children with SEN constituted a clinically heterogeneous group; however, they shared common characteristics relevant to the present study. Compared with typically developing children, they generally experienced greater difficulties in behavioural self-regulation, sustained attention, inhibitory control, behavioural flexibility, and social adaptation. These domains represent the primary targets of dog-assisted animal-assisted therapy and therefore provide a rationale for examining intervention effects separately in children with and without SEN.

Children without an official diagnosis were classified as typically developing and did not receive specialised educational support services. This distinction is important because children with SEN generally present greater difficulties in behavioural self-regulation, attention control, executive functioning, and social adaptation than their typically developing peers, which may increase the potential benefits of structured interventions such as animal-assisted therapy.

Statistical power was evaluated using G*Power 3.1. for the two primary research questions. For the first hypothesis, concerning the overall effectiveness of the intervention, power was estimated for the Group × Time interaction in a repeated measures ANOVA with two groups and two measurement occasions. For the second hypothesis, concerning differential intervention effects by child type, power was estimated for the Group × Time × Child Type interaction, corresponding to four between-subject combinations and two measurement occasions. Power calculations were conducted using α = 0.05, an assumed correlation of 0.50 between repeated measurements, and a nonsphericity correction of ε = 1.00.

The experimental group consisted of 114 participants (Table 1). Regarding gender distribution, the majority of the participants were female (73 participants, 64.0%), while males accounted for 41 participants (36.0%). The age distribution indicated that most participants belonged to the middle age categories: 30 participants (26.3%) were classified in category 6, 25 participants (21.9%) in category 5, 18 participants (15.8%) in category 4, 16 participants (14.0%) in category 7, and 14 participants (12.3%) in category 8. The youngest and oldest categories (categories 2 and 10) each included only one participant (0.9%). The distribution of type of residence was relatively balanced, with 61 participants (53.5%) living in smaller cities or villages and 53 participants (46.5%) in bigger cities or the capital. With regard to mothers’ educational attainment, the most common level was a university degree (40 participants, 35.1%), followed by a high school diploma (29 participants, 25.4%) and a college degree (22 participants, 19.3%). Vocational school education was reported for 14 participants (12.3%), primary school education (8 grades) for 8 participants (7.0%), and less than eight years of primary education for 1 participant (0.9%). A similar pattern was observed for fathers’ educational attainment: 34 participants (29.8%) reported that their fathers held a university degree, 31 (27.2%) a high school diploma, 25 (21.9%) a vocational school qualification, 14 (12.3%) a college degree, and 10 (8.8%) primary school education (8 grades). Regarding the subjective assessment of family financial status, the largest proportion of participants selected category 3 (48 participants, 42.1%), reflecting a generally adequate financial situation with careful budgeting. Category 2 was selected by 33 participants (28.9%), category 4 by 21 participants (18.4%), and category 1 by 12 participants (10.5%).

The control group consisted of 87 participants (Table 1). Regarding gender distribution, the majority of the participants were female (51 participants, 58.6%), while males accounted for 36 participants (41.4%). The mean age of the participants was 7.71 years (SD = 1.19). Regarding SEN status, 32 participants (36.8%) were classified as having special educational needs, while 55 participants (63.2%) were typically developing children. The distribution of type of residence was relatively balanced, with 46 participants (52.9%) living in bigger cities or the capital and 41 participants (47.1%) in smaller cities or villages. With regard to mothers’ educational attainment, the most common level was a university degree (27 participants, 31.0%), followed by a high school diploma (24 participants, 27.6%) and a college degree (17 participants, 19.5%). Vocational school education was reported for 12 participants (13.8%), primary school education (8 grades) for 6 participants (6.9%), and less than eight years of primary education for 1 participant (1.1%). A similar pattern was observed for fathers’ educational attainment: 24 participants (27.6%) reported that their fathers held a university degree, 25 (28.7%) a high school diploma, 18 (20.7%) a vocational school qualification, 11 (12.6%) a college degree, and 9 (10.3%) primary school education (8 grades). Regarding the subjective assessment of family financial status, the mean score was 2.69 (SD = 0.85).

### 2.2. Instruments

The study used questionnaires, which allowed us to collect data in a structured way. In our self-designed Sociodemographic Questionnaire, we formulated questions on gender, age, type of residence, parents’ highest level of education, family structure, subjectively assessed financial situation, number of siblings, special educational needs, and behaviour in educational institutions.

To assess the child’s perceived behaviour, we used the parent version of the Conners Child Behaviour Questionnaire (Conners, 1970, 1973), a widely used instrument for evaluating behavioural difficulties in children aged 3–17 years. The questionnaire contains 27 items and provides scores on four domains: oppositional behaviour problems, cognitive difficulties, hyperactivity and ADHD symptoms. Higher scores indicate greater levels of behavioural difficulties. In the present study, these scales were used to evaluate changes in behavioural functioning before and after the intervention. The Oppositional Behaviour scale assesses behaviours related to non-compliance, irritability, and difficulties following rules and instructions. The Cognitive Problems/Inattention scale reflects difficulties in attention, concentration, and task completion. The Hyperactivity scale measures behaviours associated with excessive activity, impulsivity, and restlessness. The ADHD Index provides a summary indicator of behavioural characteristics commonly associated with attention-deficit/hyperactivity disorder and is intended as a screening measure rather than a diagnostic tool.

The Strengths and Difficulties Questionnaire (SDQ) [32], designed by the English child psychiatrist Robert Goodman, was based on the Rutter parent questionnaire, which is shorter than the Child Behaviour Checklist (CBCL) version of the most commonly used behavioural and emotional problems, but needed further development. The SDQ is a short behaviour screening questionnaire for children aged 2–17 years. Several versions exist. The instrument measures the following domains: emotional symptoms (5 items), conduct problems (5 items), hyperactivity/attention deficit (5 items), peer relationship problems (5 items), and prosocial behaviour (5 items). The Conners questionnaire was used to assess ADHD-related behavioural symptoms, whereas the SDQ provides a broader screening of emotional and behavioural difficulties, including prosocial behaviour and peer relationships. Using both instruments allowed us to capture complementary aspects of children’s behavioural functioning.

### 2.3. Procedure

A total of ten weekly sessions, each lasting half an hour, of AAT were offered to participants. This decision was based on clinical experience, as no established protocols were available to determine the ideal number of sessions. Children assigned to the therapy group received ten sessions with a therapy dog, while those in the control group did not receive dog-assisted therapy. Each therapy session featured interventions focused on occupation and aimed at achieving specific goals. Activities included in these sessions are detailed in Table 2.

The intervention followed a structured developmental and behavioural approach. Activities were designed to promote attention regulation, behavioural self-regulation, cooperation, task engagement, and cognitive functioning through guided interactions with the therapy dog. Sessions incorporated elements of play-based learning, behavioural modelling, structured instruction, and positive reinforcement. The therapy dog served as a motivating and supportive partner during the activities, while the therapist guided task completion and behavioural practice. The intervention sessions were conducted by a certified animal-assisted therapist working together with a trained and certified therapy dog. The therapist had professional experience in educational and developmental interventions with children and had completed specialised training in animal-assisted interventions. All dog-assisted therapy sessions were conducted by the same certified AAT team.

The control group did not receive any intervention from the research team during the study period and continued only with their usual educational and developmental services. Therefore, therapist-related variability across intervention and control conditions could not be modelled statistically, as there were no different therapists delivering different study conditions. Children assigned to the control group continued to receive their usual educational and developmental services provided by their schools or institutions but did not participate in the dog-assisted therapy programme during the study period. No additional intervention was introduced by the research team.

All participants completed the baseline assessment. Post-intervention data were collected after the completion of the ten-session intervention period. The analyses were conducted on all participants with available pre- and post-intervention data.

### 2.4. Statistical Analysis

The study used a parallel-group randomised controlled trial design. Children were randomly assigned to either the experimental group that received AAT (AAT) or the control group that received no such intervention.

Randomisation was performed at the individual child level using a computer-generated random allocation sequence with an intended 1:1 allocation ratio. Allocation was completed before the start of the intervention by a member of the research team who was not involved in delivering the intervention sessions. Simple randomisation without blocking or stratification was used. Consequently, although the intended allocation ratio was 1:1, an unequal distribution of participants between the intervention (n = 114) and control (n = 87) groups occurred by chance. No post-randomisation reassignment or selective allocation of participants was performed, and all randomised participants were analysed according to their assigned study group.

The a priori power analysis indicated that a total sample of 128 participants would provide 80% power to detect a medium-sized Group × Time interaction (f = 0.25, α = 0.05). The final sample of 201 participants therefore exceeded the required sample size for the primary hypothesis.

Data were summarised in Excel spreadsheets and analysed using IBM SPSS 22.0 (IBM Corp., Armonk, NY, USA) and Jamovi Version 2.3.28. Descriptive statistics and data management procedures were conducted in SPSS, whereas inferential analyses were performed and verified using Jamovi. The use of two software packages served as a cross-check of the statistical results. Descriptive statistics (means and standard deviations) were calculated for all outcome variables at baseline and post-intervention for both groups. Baseline differences between groups were examined using independent-samples tests. Prior to the analyses, the distribution of the variables was examined using the Shapiro–Wilk test and visual inspection of Q-Q plots. Several outcome variables deviated significantly from normality; therefore, non-parametric Wilcoxon signed-rank tests were used to compare pre- and post-intervention scores within groups. To evaluate the effectiveness of the intervention, mixed-design analyses of variance (mixed ANOVA) were conducted with time (pre-test vs. post-test) as the within-subject factor and group (experimental vs. control) as the between-subject factor. Although non-parametric Wilcoxon signed-rank tests were used for within-group comparisons, the mixed ANOVA was retained for the examination of Group × Time interaction effects. Mixed ANOVA is generally considered robust to moderate departures from normality, particularly in samples of this size (N = 201), and provides a direct test of interaction effects that is not readily available through standard non-parametric procedures. Effect sizes for the mixed ANOVA analyses were reported as partial eta squared (ηp^2^). Interpretation followed Cohen’s (1988) guidelines, where ηp^2^ = 0.01 indicates a small effect, ηp^2^ = 0.06 a medium effect, and ηp^2^ = 0.14 a large effect. In addition, baseline values were included as covariates in supplementary analyses to account for potential baseline differences between groups. Because multiple subscales of the Conners and SDQ were analysed, the results should be interpreted cautiously. No formal correction for multiple comparisons was applied, and therefore the findings should be considered exploratory.

## 3. Results

Table 3 shows the characteristics of the intervention and control groups regarding the psychological variables tested in the research.

### 3.1. Intervention Effects: Group × Time Interaction Analyses

To evaluate the primary hypothesis, mixed-design repeated measures ANOVAs were conducted to examine whether behavioural changes over time differed between the intervention and control groups. The primary test of intervention effectiveness was the Group × Time interaction.

The overall repeated measures ANOVA did not reveal a significant Group × Time interaction (F(1,197) = 0.99, *p* = 0.322), indicating that behavioural changes over time did not differ significantly between the intervention and control groups. Likewise, the Group × Time × Outcome interaction was not significant (F(3,591) = 0.75, *p* = 0.525), suggesting that the pattern of change across the behavioural outcomes was comparable in the two groups.

Consequently, the repeated measures ANOVA did not provide overall evidence that the intervention resulted in greater behavioural improvements than those observed in the control condition. Therefore, the primary hypothesis was not supported by the omnibus interaction analyses (see Table 4).

### 3.2. Differential Effects in Children with Special Educational Needs

To examine the second hypothesis, mixed-design repeated measures ANOVAs were extended to include child type (children with special educational needs [SEN] vs. typically developing children) as an additional between-subjects factor. The critical test of this hypothesis was the Group × Time × Child Type interaction.

No significant Group × Time × Child Type interactions were observed for any of the behavioural outcomes (all *p* > 0.05). These findings indicate that the intervention did not produce significantly different behavioural changes in children with SEN compared with typically developing children. Therefore, the second hypothesis was not supported.

### 3.3. Within-Group Changes (Supplementary Analyses)

Within-group analyses were conducted using Wilcoxon signed-rank tests to describe pre- to post-intervention changes separately in the intervention and control groups. These analyses are presented as supplementary evidence and should be interpreted descriptively. The primary evaluation of intervention effectiveness is based on the Group × Time interaction analyses reported above.

Within the intervention group, parent-reported Conners Oppositional, Cognitive Problems/Inattention, and Hyperactivity scores showed significant reductions from baseline to post-intervention. Significant improvements were also observed for Conduct Problems on the SDQ, whereas the remaining SDQ subscales did not change significantly.

Within the control group, significant reductions were likewise observed for parent-reported Conners Oppositional and Hyperactivity scores, while no significant changes were found for the remaining Conners or SDQ outcomes.

Because improvements were observed in both groups for some behavioural outcomes, these within-group findings should not be interpreted as evidence of intervention effectiveness. Rather, they provide descriptive information about changes over time within each group. The effectiveness of dog-assisted therapy is determined by the Group × Time interaction analyses presented in Section 3.1.

**Figure 2:**
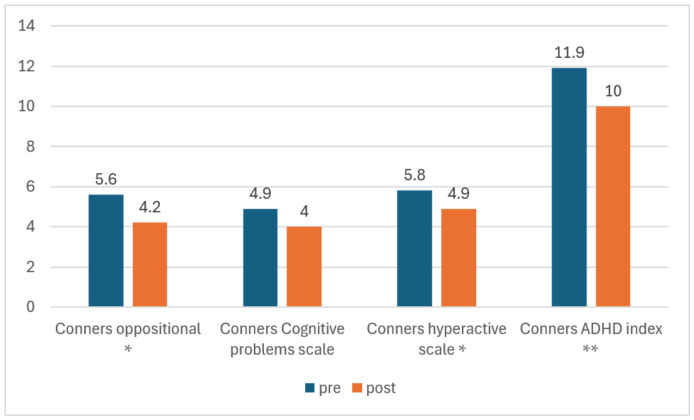
Conners scale pre-and post-test results for the experimental group. * *p* < 0.05; ** *p* < 0.01.

Regarding the experimental group, we looked at whether there was a difference between the experimental group’s pre-and post-test results. The Descriptive Analysis shows a significant decrease in mean scores on the Conners scales, particularly in the oppositional, cognitive and hyperactive domains. The pre-intervention Conners Oppositional mean score was 5.693, which decreased to 4.281 after the intervention, showing a significant difference and, therefore, a significant improvement in oppositional behaviour. These findings indicate that parents reported lower levels of oppositional behaviour following the intervention. Similarly, the mean score of Conners ADHD decreased from 11.982 to 10.026, which is a significant result, i.e., Parent-reported ADHD-related symptoms and hyperactivity scores were lower following the interventions (Figure 2).

**Figure 3:**
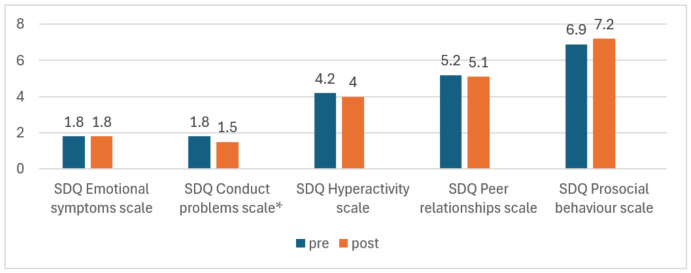
SDQ scale pre-and post-test results for the experimental group. * *p* < 0.05.

Regarding the Strength and Difficulties Questionnaire, only the Behavioural Problems scale showed significant improvements (Figure 3). In this case, we could see that parents reported fewer behavioural problems after the intervention. The other subscales showed a mild decrease as well, except for the emotional symptoms.

### 3.4. The Results of the Research for the Control Group

These analyses were also carried out for the control group. In this case, a change was also observed for the Conners Opposition and Hyperactivity scales, where statistically significant changes were found, i.e., some improvement was also observed in the control group. These results suggest that the intervention was not the only determining factor for the subscales studied. Other factors may have influenced the results, such as the individual characteristics of the participants, environmental influences and social interactions. In contrast, there were no significant differences in any of the SDQ subscales, suggesting that the intervention led to significant changes in these areas, as the experimental group showed improvement.

**Figure 4:**
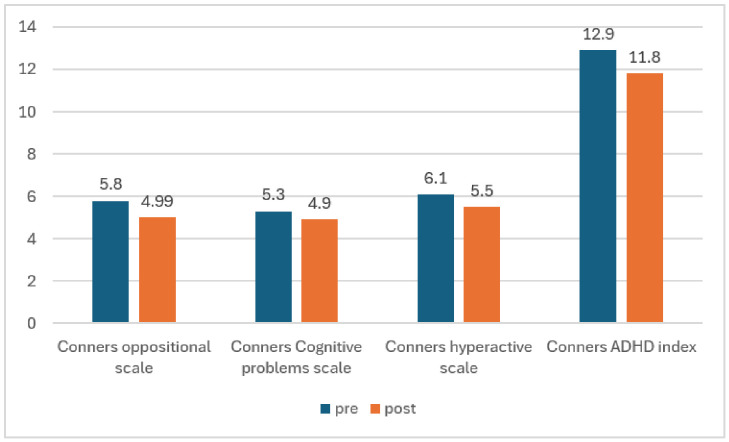
Conners scale pre-and post-test results for the control group.

The Conners scale is used to assess children’s ADHD and behavioural problems. In the analysis, we observed beneficial changes in different domains, particularly in the oppositional, cognitive and hyperactive subscales, based on pre-and post-test comparisons (Figure 4). The Conners’ Oppositional mean score decreased, which shows a significant difference, indicating that children’s oppositional behaviour improved significantly, including greater cooperation in the preschool and home environments. Similarly, the mean Conners ADHD score also decreased, a change that also shows positive signs for attention deficit and hyperactivity, with a reduction in ADHD symptoms, demonstrating the effectiveness of AAT.

**Figure 5:**
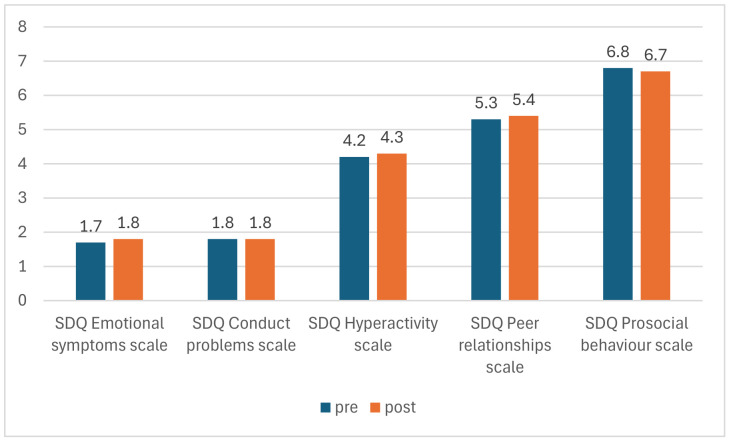
SDQ scale pre-and post-test results for the control group.

Although the Conners scale scores showed clear improvements, only the Scale of Behavioural Problems showed significant improvements on the Strength and Difficulties Questionnaire (SDQ) (Figure 5). This suggests that the children did not show the same level of change in other aspects of their behaviour as the Conners scale scores indicated, but it is encouraging that there was also an improvement in behavioural problems.

**Figure 6:**
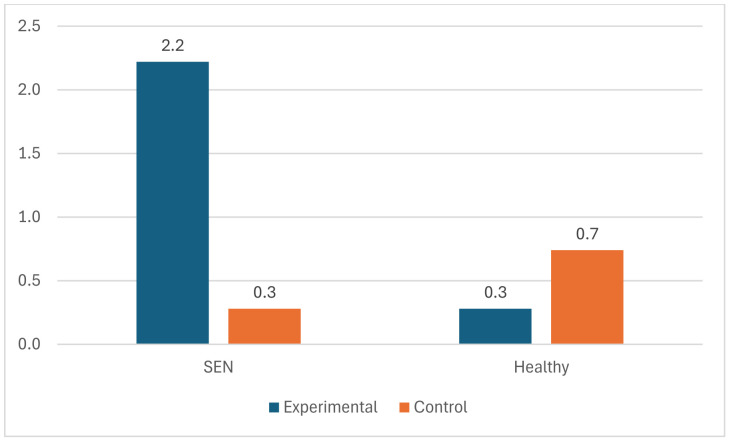
Differences between typically developing and SEN children.

Compared with typically developing children, those with SEN showed substantially greater mean improvement following the intervention. The average change for the experimental SEN group was 2.22, which indicated a significant improvement and suggested that the SEN group showed greater parent-reported improvements than the typically developing group (Figure 6). In contrast, the average change in the experimental typically developing group was 0.286, which also showed an improvement, but not to the same extent as in the SEN group.

Another aspect is the comparison of control groups. The average change in the control SEN group was 0.333, which was lower than the performance of the experimental SEN group, but the average change in the control typically developing group was 0.741. This latter result may suggest that the typically developing control group was operating under more favourable conditions, which resulted in the higher rate of change. These findings suggest that children with SEN may have derived greater benefit from the intervention than their typically developing peers. However, these subgroup comparisons should be interpreted cautiously, as no statistically significant Group × Time × Child Type interaction was observed in the repeated measures ANOVA (F(3,591) = 0.75, *p* = 0.53). Thus, although the descriptive trends appear meaningful, they do not provide conclusive statistical evidence that the intervention effect differed according to SEN status.

Overall, the findings suggest that AAT may be particularly beneficial for children with SEN. However, because the subgroup interaction was not statistically significant, these results should be interpreted as preliminary and confirmed in future studies with larger samples.

## 4. Discussion

The present randomised controlled trial examined whether dog-assisted animal-assisted therapy (AAT) resulted in greater improvements in children’s behavioural functioning than those observed in a control group and whether these effects differed between children with special educational needs (SEN) and typically developing children. Contrary to our primary hypothesis, the mixed-design ANOVA did not demonstrate significant Group × Time interaction effects, indicating that behavioural changes over time did not differ significantly between the intervention and control groups. Likewise, no significant Group × Time × Child Type interactions were observed, suggesting that children with SEN did not benefit significantly more from the intervention than their typically developing peers.

Although within-group analyses showed reductions in parent-reported oppositional behaviour, cognitive problems/inattention, hyperactivity, ADHD symptoms, and conduct problems in the intervention group, similar improvements were also observed for some outcomes in the control group. Consequently, these within-group findings cannot be interpreted as evidence of intervention effectiveness. Instead, the randomised comparison indicates that the observed behavioural improvements cannot be confidently attributed to the dog-assisted intervention alone.

Several methodological factors may have contributed to the limited support for the expected intervention effects. First, although the study included more than 200 participants, the statistical power may have been insufficient to detect relatively small Group × Time interaction effects, particularly in subgroup analyses involving children with SEN. Second, behavioural outcomes were assessed exclusively using parent-report questionnaires. Although parent reports provide valuable information about children’s everyday behaviour, they may be influenced by expectancy effects and may be less sensitive to behavioural changes occurring primarily within the school environment. Third, the intervention consisted of ten sessions, which may have been insufficient to produce measurable behavioural changes detectable by standardised questionnaires.

At the same time, the present findings should not be explained solely by methodological considerations. It is also possible that the effectiveness of dog-assisted AAT for improving behavioural functioning is more modest than suggested by previous studies. Many earlier investigations have reported improvements in attention, behavioural regulation, emotional functioning, and quality of life following dog-assisted interventions [17]. However, a considerable proportion of these studies relied on pre- and post-intervention comparisons without randomised control groups or included relatively small samples and heterogeneous intervention protocols. Consequently, some previously reported improvements may partly reflect natural developmental changes, non-specific therapeutic effects, or repeated assessment rather than intervention-specific effects [33].

Nevertheless, several theoretical mechanisms may explain why AAT has the potential to influence behavioural functioning. Interaction with therapy animals has been associated with improved behavioural self-regulation, attentional control, and emotional security [19,20]. The unconditional acceptance provided by animals may strengthen children’s self-confidence and emotional stability, thereby facilitating concentration and reducing behavioural resistance [34,35]. Furthermore, structured therapy sessions require children to follow instructions, wait their turn, regulate their behaviour, and sustain attention while interacting with the therapy dog, thereby providing repeated opportunities to practise executive functions and inhibitory control [36,37].

Similarly, previous research suggests that therapy dogs may facilitate children’s engagement in structured therapeutic activities by increasing motivation, perceived safety, and willingness to participate [22,23]. Interaction with therapy animals has also been linked to stronger emotional bonding and improved social interaction, while experiential learning during dog-assisted activities may promote active participation in therapeutic tasks [38,39]. These mechanisms remain theoretically plausible even though the present randomised comparison did not demonstrate statistically significant superiority of the intervention over the control condition.

Although descriptively larger improvements were observed among children with SEN, the Group × Time × Child Type interaction was not statistically significant. Therefore, the present study does not provide sufficient evidence that dog-assisted AAT is more effective for children with SEN than for typically developing children. Nevertheless, because children with SEN frequently experience greater difficulties in behavioural self-regulation, attention, and social functioning, future studies with larger samples and sufficient statistical power should continue to investigate whether specific diagnostic groups benefit differentially from this type of intervention [28].

When comparing children with SEN and typically developing children, larger improvements were observed among children with SEN. Compared to the mean change score in the experimental SEN subgroup, the corresponding change scores in the control group were substantially smaller. These findings suggest that children with SEN may benefit more strongly from dog-assisted animal-assisted therapy than their typically developing peers. One possible explanation is that children with SEN often begin the intervention with greater difficulties in attention regulation, behavioural control, and social functioning, leaving more room for improvement. The structured interaction with the therapy dog may provide additional motivation, emotional security, and opportunities to practise self-regulation skills that are particularly relevant for this population. However, these subgroup findings should be interpreted cautiously because the study was not specifically powered for detailed analyses of individual diagnostic categories.

One of the major strengths of the present study is its randomised controlled design, which provides a more rigorous evaluation of intervention effects than uncontrolled pre-post studies. By comparing behavioural changes over time between intervention and control groups, the study reduces the risk of attributing naturally occurring developmental changes or repeated testing effects to the intervention itself. Future research should include longer intervention periods, larger samples, multi-informant outcome assessments (parents, teachers, and independent observers), and longer follow-up periods to determine under which conditions dog-assisted AAT may produce clinically meaningful behavioural improvements.

### Limitations

However, it is also necessary to mention the limitations of the research. Although the sample included participants from both Hungary and Romania and represented a range of socioeconomic and educational backgrounds, the study was conducted exclusively within two Central European countries. Therefore, the findings may not generalise to children from other cultural, educational, and healthcare contexts.

Although significant improvements were observed in several Conners subscales, including oppositional behaviour, cognitive problems, hyperactivity, and the ADHD index, as well as in the SDQ Conduct Problems scale, no significant changes were found in emotional symptoms, peer relationship problems, prosocial behaviour, or SDQ hyperactivity scores. These findings suggest that the intervention may be more effective for specific behavioural domains than for broader emotional and social outcomes.

One must mention that the study assessed outcomes immediately after completion of the intervention, and no follow-up measurements were conducted. Consequently, it remains unclear whether the observed improvements in behavioural functioning were maintained over time. Longitudinal studies are needed to determine the durability of the effects of AAT.

Another limitation concerns the improvements observed in some measures within the control group. Significant reductions were also found in oppositional behaviour, hyperactivity, and ADHD-related symptoms among children who did not participate in the intervention. This indicates that developmental maturation, educational experiences, family influences, or other environmental and social factors may have contributed to behavioural changes during the study period. Therefore, the effects attributed to AAT should be interpreted with caution.

Furthermore, behavioural functioning was assessed exclusively through parent-reported questionnaires. Although parent ratings provide valuable information regarding children’s everyday behaviour, reliance on a single informant may introduce reporting bias. Future studies would benefit from incorporating multiple sources of information, including teacher reports, clinician ratings, direct behavioural observations, and objective measures of attention and self-regulation.

The study included children with special educational needs and typically developing children; however, children with more severe intellectual disabilities or severe autism spectrum disorder were excluded. Consequently, the results cannot be generalised to all populations of children with special educational needs.

An additional limitation is that potential associations between sociodemographic characteristics and intervention outcomes were not examined in the present study. Factors such as age, gender, place of residence, parental educational attainment, and family socioeconomic circumstances may influence behavioural functioning and responsiveness to the intervention. Future studies should investigate whether these variables moderate the effectiveness of AAT.

## 5. Conclusions

The present randomised controlled study provides evidence that dog-assisted AAT may contribute to improvements in selected aspects of behavioural functioning among preschool and primary school children. The findings suggest that AAT may contribute to improvements in behavioural functioning, although improvements were also observed in the control group and the results should therefore be interpreted cautiously. Dogs’ roles as emotional supporters and social facilitators can be extremely important in children’s development, providing them with opportunities to learn social interaction, communication, and empathy. At the same time, the findings indicate that the effects of the intervention were not uniform across all behavioural and emotional domains. No significant improvements were observed in emotional symptoms, peer relationship problems, or prosocial behaviour. Furthermore, some improvements were also detected in the control group, suggesting that factors other than the intervention itself may have influenced behavioural development during the study period. The present findings should be interpreted with caution because behavioural outcomes were assessed exclusively through parent-reported questionnaires. As parents were aware of their children’s participation in the intervention, expectancy effects and reporting biases cannot be excluded. Future studies should incorporate teacher reports, direct behavioural observations, and objective measures of attention and self-regulation to provide a more comprehensive evaluation of intervention effectiveness.

Still, the practical implications of this research are significant on several levels, particularly in education, therapy and support for children’s behavioural and emotional well-being. AAT was effective in improving children’s cognitive functioning and attention, which may be particularly useful in school settings where children have attention deficit hyperactivity disorder or ADHD. This suggests that educational institutions can integrate animal-assisted sessions into learning programmes. The demonstrated effect of reducing oppositional behaviour may promote classroom discipline and cooperation. Such programmes can contribute to the integration of children with behavioural difficulties. AAT has also been shown to be particularly effective for children with SEN, improving their social interactions, behaviour and attention, suggesting that the therapy could be used more widely in special schools and institutions. Interaction with dogs helps children to learn empathy and social norms. This is particularly important for children who have difficulties with social behaviour and emotional attachment. The results of this research underline the importance of the therapeutic effects of animals, which may encourage policymakers and practitioners to support the broader use of animal-assisted therapies. These findings support the use of structured animal-assisted interventions to improve self-regulation and reduce disruptive behaviours in preschool and primary school children, particularly those with SEN. Future research should focus on long-term follow-up assessments, the inclusion of multiple informants and objective outcome measures, and the examination of intervention effects across more diverse cultural contexts and clinical populations. Such studies would help clarify the mechanisms underlying the observed improvements and strengthen the evidence base for animal-assisted interventions in childhood development.

## Figures and Tables

**Table 1:** Sociodemographic characteristics of the groups.

Variable	Intervention Group n = 114	Control Group n = 87
Age, M ± SD	7.58 ± 1.24	7.71 ± 1.19
Gender, n (%) female	73 (64.0%)	51 (58.6%)
SEN status, n (%)	37 (32.5%)	32 (36.8%)
Residence type, n (%) urban	53 (46.5%)	46 (52.9%)
Mother’s education, university degree n (%)	40 (35.1%)	27 (31.0%)
Father’s education, university degree n (%)	34 (29.8%)	24 (27.6%)
Subjective family financial status (1–4), M ± SD	2.80 ± 0.89	2.69 ± 0.85

**Table 2:** The description of the dog-assisted therapy sessions and their developmental goals.

No.	Description of the Session	Developmental Goals
1	Getting to know the dog. - talk about the dog the dog	Socialisation, communication
2	Colour identification, pairing - children take the coloured dog off the harness and put it in the corresponding coloured house. - take a picture from the basket that the dog has brought and place it on the appropriate colour palette	- developing visual perception - developing creative thinking - developing language skills
3	Shadow Search - children each take a picture from the basket and look for the dog’s shadow on the poster on the board. - you need to find the shadow of a particular dog from among several shadows	- developing visual perception - developing resilience - developing language skills
4	Imitate the dog - when the dog sits, the children sit; when the dog stands, they stand; when the dog high-fives, they high-five, etc.	- developing observation and imitation skills - body pattern development - body image development - developing spatial orientation
5	Control the dog - on the ground are rings; each ring has a picture showing what the dog is to do, to stand, sit or lie down - the children perform the task one by one, using hand signals and words to guide the dog and reward the dog at the end of the task	- developing attention - developing cooperation - improving communication - developing self-confidence
6	Pairing - as the therapist walks the dog past the children, they must find and remove the picture from the cards clipped to the dog’s harness that matches the picture in their hand	- attention, memory development - improving the ability to maintain concentration - developing perseverance - developing critical thinking
7	Activity. Selection of seasons - the therapist and the dog lie down on the floor next to the pictures and wait for the children to place the objects related to the different seasons in the basket	- developing attention and flexible thinking - improving the ability to maintain concentration - developing critical thinking
8	Obstacle course - there are buoys to go around, sensory pads to go over, a tilting bridge to go over, and the therapist and the dog to show how to go over the obstacle course.	- developing attention coordination of movements - improving the ability to maintain concentration - developing cooperation
9	Moving truck - a track with different movements: elements of the movement clay (rabbit, bird, bear, frog, etc.), a hoop in a buoy.	- developing attention and coordination of movements - improving the ability to maintain concentration - developing cooperation
10	Flower collection - colourful flowers are placed on the ground. - one by one, children approach the dog, find the flower that matches the pattern, and place it on the dog’s leash, paying attention to the order of the colours.	- developing attention and coordination of movements - improving the ability to maintain concentration - developing cooperation

**Table 3:** Psychological characteristics of the groups.

Variable	Intervention Group n = 114	Control Group n = 87
Conners Oppositional, M ± SD	5.69 ± 2.81	5.82 ± 2.76
Conners Cognitive Problems/Inattention, M ± SD	4.91 ± 2.44	4.99 ± 2.51
Conners Hyperactivity, M ± SD	5.81 ± 2.72	6.07 ± 2.83
Conners ADHD Index, M ± SD	11.98 ± 4.52	12.93 ± 4.76
SDQ Emotional Symptoms, M ± SD	1.81 ± 1.56	1.72 ± 1.49
SDQ Conduct Problems, M ± SD	1.82 ± 1.37	1.81 ± 1.35
SDQ Hyperactivity, M ± SD	4.22 ± 2.11	4.24 ± 2.06
SDQ Peer Problems, M ± SD	5.24 ± 2.09	5.31 ± 2.02
SDQ Prosocial Behaviour, M ± SD	6.94 ± 1.98	6.82 ± 1.91

**Table 4:** Statistical outcomes.

Outcome	Group × Time F (df1, df2)	*p*	ηp^2^	Interpretation
Conners Oppositional	F(1,198) = 0.22	0.643	0.001	negligible
Conners Cognitive Problems/Inattention	F(1,198) = 1.53	0.218	0.008	negligible
Conners Hyperactivity	F(1,198) = 1.22	0.271	0.006	negligible
Conners ADHD Index	F(1,198) = 2.36	0.126	0.012	small
SDQ Emotional	F(1,198) = 0.13	0.762	0.001	negligible
SDQ Conduct	F(1,198) = 1.42	0.289	0.001	negligible
SDQ Hyperactivity	F(1,198) = 1.21	0.193	004	negligible
SDQ Peer Problems	F(1,198) = 1.67	0.253	005	negligible
SDQ Prosocial	F(1,198) = 1.18	0.224	003	negligible

## Data Availability

Data are available only on request due to ethical restrictions. For further information, please contact the corresponding author, Éva Zita Balogh (kovacs.karolina@arts.unideb.hu).

## Abbreviations

The following abbreviations are used in this manuscript:

AAT	Animal-assisted therapy
SDQ	Strength and Difficulties Questionnaire
SEN	Special education needs
